# Cattle Management for Dairying in Scandinavia’s Earliest Neolithic

**DOI:** 10.1371/journal.pone.0131267

**Published:** 2015-07-06

**Authors:** Kurt J. Gron, Janet Montgomery, Peter Rowley-Conwy

**Affiliations:** Department of Archaeology, Durham University, Durham, United Kingdom; Museo Nazionale Preistorico Etnografico 'L. Pigorini', ITALY

## Abstract

New evidence for cattle husbandry practices during the earliest period of the southern Scandinavian Neolithic indicates multiple birth seasons and dairying from its start. Sequential sampling of tooth enamel carbonate carbon and oxygen isotope ratio analyses and strontium isotopic provenancing indicate more than one season of birth in locally reared cattle at the earliest Neolithic Funnel Beaker (EN I TRB, 3950-3500 cal. B.C.) site of Almhov in Scania, Sweden. The main purpose for which cattle are manipulated to give birth in more than one season is to prolong lactation for the production of milk and dairy-based products. As this is a difficult, intensive, and time-consuming strategy, these data demonstrate complex farming practices by early Neolithic farmers. This result offers strong support for immigration-based explanations of agricultural origins in southern Scandinavia on the grounds that such a specialised skill set cannot represent the piecemeal incorporation of agricultural techniques into an existing hunter-gatherer-fisher economy.

## Introduction

The appearance of agriculture caused massive social and economic changes throughout Europe and the world. Despite this, relatively little is known about the nature of early animal husbandry. This is particularly true in southern Scandinavia during the first five hundred years of the Neolithic (Early Neolithic I, Funnel Beaker Culture, EN I TRB, ca. 3950–3500 B.C.). We know that domestic animals were present, but we know nothing of their management. In part, this owes to the scarcity of the material, which in most cases is limited to a small number of faunal remains from each individual site or to bones in poor condition [1–5].

The adoption of an agricultural way of life in the region has been a major research focus. Most research has attempted to tackle the question directly; that is, endeavouring to pinpoint explanatory factors at the point of transition or across the transition [6–14]. In practice this has meant focusing on the process and timing of Neolithisation, similarities and dissimilarities between the Neolithic and the proceeding Mesolithic, and which climatic or subsistence changes coincide with the arrival of agriculture. Particularly lacking is basic information of how the earliest domesticated plants and animals were managed by the inhabitants of the region.

**Fig 1:**
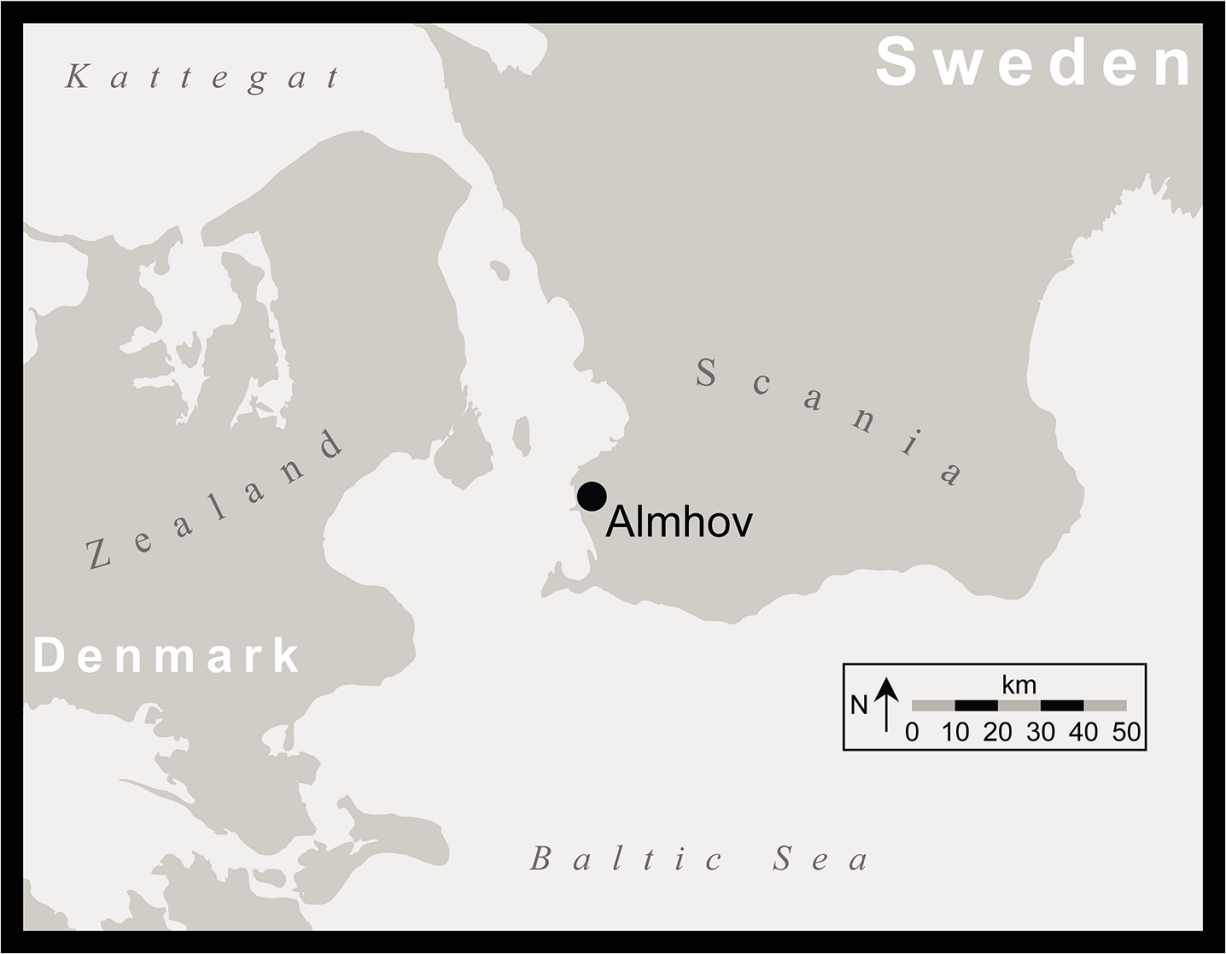
The location of Almhov in Scania.

In this study, we report data on birth seasonality and provenance of domesticated cattle (*Bos taurus*) deriving from Almhov, a Neolithic site in Scania, southern Sweden, located near the modern city of Malmö (Fig 1). The sample here represents one of the largest, securely dated assemblages deriving from the EN I TRB, and one of the only sites to yield remains of more than a few domestic cattle. Sample size is modest, but material dating from this period is extremely rare, and the Almhov sample currently represents the only opportunity to investigate cattle husbandry at a single site in the region. Our successful determination of basic information concerning birth seasonality in cattle represents the first data of its kind from this crucial early period of the Scandinavian Neolithic. While the presence of dairy products at this date is established in Sweden [15], our data illustrate how cattle were manipulated to maximize milk yields as a primary mode of agricultural production at the very start of farming.

### Neolithisation and Cattle Husbandry

The transition to agriculture in southern Scandinavia has been the focus of extensive scholarship [6–14]. This is because the region, encompassing all of Denmark, southern Sweden, and the western Baltic, witnessed the introduction of agriculture at around 3950 cal. B.C. only after retaining a predominantly hunter-gatherer economy for the preceding millennium, despite the presence of Linearbandkeramik (LBK) and Rössen farmers just to the south. When agriculture finally did arrive, it was accompanied by huge changes. The largest hunter-gatherer settlements in the Late Mesolithic were on the coasts, and most were seasonally occupied. These were abandoned in favour of permanent inland farming settlements. Major changes in lithic and ceramic technology probably reflect the influence of post-Rössen farmers to the south. New mortuary practices involved burial in earthen long barrows (burial mounds), also similar to examples further south and west in Europe [16]. Large-scale excavations are rarely undertaken around such long barrows, but in some cases they have revealed extensive areas of contemporary pits. These do not appear to be domestic settlements, but may represent temporary communal gatherings bringing together people from residential sites in the surrounding region. The Almhov site is one of these, with many pits grouped near several long barrows [17].

While there is a lack of consensus concerning the causes of the transition from the Mesolithic Ertebølle Culture (EBK, 5400-3950 cal. B.C.) to the Neolithic Funnel Beaker Culture (TRB, 3950-2800 cal. B.C.), the timeline of the arrival of the Neolithic is largely agreed upon [11, 13]. Except for the dog (*Canis familiaris*), there is no convincing evidence for domesticated plants and animals in southern Scandinavia before ca. 3950 cal. B.C. [10, 16].

However, the question of exactly what happened at the transition remains elusive. A major problem is the lack of clarity about the contribution plant cultivation made to human subsistence in the EN I. Domesticated plant foods cannot be convincingly shown to have been a significant contributor to the diet during this period [10, 18], and widespread forest clearance is not evident until the Middle Neolithic (MN) [19]. Further, domesticated animals are present at or around 3950 cal. B.C., but their role in human subsistence economies remains unresolved until the start of the MN around 3300 cal. B.C. It is only later that agricultural activities and settlements become more visible, and the residents of southern Scandinavia can be considered “fully” Neolithic [10, 18].

The early TRB (EN I) faunal assemblages usually are very small, poorly preserved, and/or difficult to date [2–4], which in part explains the dearth of knowledge concerning animal husbandry in this period. Previous applications of isotope ratio analysis into husbandry strategies are limited to a single comparative study of the diets of Holocene cattle and aurochs (*Bos primigenius*) from Denmark that investigated the types of environments utilised by the wild and domestic bovids [20]. Even basic aspects of the life histories of domestic species, such as in which season domestic animals were born, are completely unknown and have only been assumed.

The natural assumption is that both wild cattle and early domestic cattle would calve only once a year. The natural breeding rhythm of cattle is not known as true wild cattle do not exist and the last survivors of the formerly widespread wild aurochs went extinct in Poland in the seventeenth century. However, some observations made prior to extinction recorded mostly spring seasonal births; occasional autumn calves died over their first winter [21]. This is similar to what is observed in European Bison (*Bison bonasus*), for which birth season data are only available from relict provisioned populations which give birth largely between May and July and only occasionally later in the year [22]. Feral cattle raised outdoors with minimal management largely give birth seasonally, in the spring [23–25] when most fodder is available. Some feral populations do give birth year-round, but these are provisioned with fodder by humans during the winter [26], and autumn and winter calves have poor survivability [23]. Furthermore, in experiments where winter provisioning of feral cattle was discontinued, within a few years the calving season became more restricted [21]. Overall, there is a strong tendency towards birth seasonality [24] to coincide with the greatest availability of feed.

Under human manipulation, dairy herds can be calved year-round, but cows cannot lactate year-round, and require a drying-up period of usually around two months between lactations to allow recuperation of the udder. Winter calving can be advantageous, as properly fed winter-calving dairy cattle may produce more milk than their spring or summer counterparts [27]. However, suitable food such as hay or leaves must be prepared and stored prior to the winter, which is a lean time in terms of suitable fodder. Milk productivity is not constant, with a steady decrease in milk production after the first few months postpartum [27]. In a dairy or beef herd calved seasonally, milk availability will also be a markedly seasonal resource. In a northern temperate environment today, dairy cattle giving birth seasonally can usually be milked from around May to September [28]. The owners of such a herd would therefore have no fresh dairy in autumn, winter, or early spring.

Traditional cattle farming in Scandinavia was characterised by seasonal peaks of production and seasonal births of animals [28–30]. In Denmark, the shift to year-round dairying was only seen in the context of a shift to an export-based economy in the late nineteenth century [28] and beef cattle in Sweden even today give birth in the spring [31]. It is fair to say that for almost all of its agrarian history, seasonal births of cattle in Scandinavian farming were the rule rather than the exception.

### Carbon, Oxygen, and Strontium Isotopes in Tooth Enamel

Cattle are a particularly good indicator of human strategies for stock rearing, predominantly because they can breed at any point in the year [32]. Unlike sheep (*Ovis aries*), for example, whose breeding is controlled at least in part by photoperiod, the reproduction of cattle can be manipulated by farmers depending on the purpose for which they are raised [33].

The enamel crowns of cattle teeth develop either prior to birth or after parturition, with the exception of the first molar (M1), whose development proceeds both *in* and *ex utero* [34]. Final enamel calcification proceeds from the unworn tooth’s crown to its enamel-root junction (ERJ) over a period of several months to a little over a year, depending on the molar [34–35]. Shortest calcification timing is in the M1, taking about six and a half to seven and a half months; in the second molar (M2) it takes about a year, and in third molars (M3) a little over a year [34–36]. During molar formation, seasonal variation in the isotope composition of ingested water is recorded, which in turn reflects seasonal variation in δ^18^O in local rainfall [37]. In Sweden, seasonal variation in rainwater δ^18^O reaches an annual minimum between mid-January and the beginning of March with a corresponding peak in the summer months. In Scania, the minimum usually occurs in the middle of February [38]. When sampled along the direction of enamel mineralisation, a sinusoidal curve of variation in δ^18^O values is obtained (S1 Fig). In enamel, the signal is both dampened and time-shifted relative to the ingested water and therefore the environmental signal [39]. However, if more than one animal is sampled in this fashion, variation in birth season can be estimated [35, 40].

δ^13^C values in tooth enamel carbonate (hydroxylapatite) of herbivores reflect the protein, carbohydrates, and fats in the diet of the animal [41]. When sampled sequentially as for δ^18^O, enamel carbonate records these components of the diet of the animal as the tooth mineralises. First molars, as they span both ante- and post-parturition periods in the cow’s life [34, 42], record significant changes in the diet and record the animal’s transition from digestion through rumination *in utero*, to non-rumination at birth due to the incomplete development of the rumen, and then rumination again as the cow grows [35]. Second and third molars should record almost entirely digestion by rumination, as they start to mineralise after the animal is born [34].

Stable isotope ratio analyses of strontium in tooth enamel and bone have proven an important tool for understanding the movement of humans and animals across landscapes and for identifying possible regions of birth [43–44]. Recent applications have demonstrated the utility of the method in southern Scandinavia with domestic cattle [45], and, importantly for this study, established the expected ranges of values for the study region of Scania as well as for the majority of southern Scandinavia [46–48]. Given that tooth enamel formation occurs at a particular point in an animal’s life, the local strontium isotope ratio is deposited into the animal’s enamel during this period and can be used to investigate where the animal spent the months during enamel formation. The bedrock geology of southern Scandinavia has yielded a small range of ^87^Sr/^86^Sr values in tooth enamel owing to the rather homogenous end moraine found across the region [47]. The cattle born in southern Scania or elsewhere in southern Scandinavia should have ^87^Sr/^86^Sr values between 0.7090 and 0.7108.

## Materials and Methods

The Swedish site of Almhov (Fig 1) was excavated in 2001 and 2002 in advance of the City Tunnel Project (*Citytunnelprojektet*), a major infrastructure development undertaking aimed at improving the rail connections between the centre of Malmö and the Öresund bridge, which connects southern Sweden and Denmark. The site is located just east of the confluence point of the main rail line between Copenhagen and Malmö and the E20 motorway, which join together to cross the Öresund bridge. Occupation is dated between the end of the Mesolithic and the middle Neolithic, with the majority of dates falling in the EN I TRB [17]. The curating institution of the Almhov material, the Malmö Museum, Malmö, Sweden gave permission for these analyses. Malmö Museum site and specimen identification numbers are listed in Table 1.

**Table 1:** Teeth sampled.

**Tooth Number**	**2**	**3**	**4**	**5**	**6**	**7**	**8**	**9**	**10**	**11**	**35**
**Animal Number**	1	2	2	3	3	4	4	5	5	5	6
**MHM Site Number**	12875	12875	12875	12875	12875	12875	12875	12747	12747	12747	12875
**MHM Number**	213965	213856	213856	213904	213904	213846	213846	1055	1055	1055	213904
**Feature**	35862	19049	19049	25594	25594	19049	19049	6	6	6	19049
**Element**	M1	dP4	M1	dP4	M1	M1	M2	dP4	M1	M2	M1
**Side**	dx	dx	dx	dx	dx	dx	dx	dx	dx	dx	sn
**Wear**	f	j/k	d	f	b	f	a	j/k	e	a	c
**DE (mm)**	31.7		33.1			30.4	35.1				33.2
**FD (mm)**	7.17		8.5		10.5	4.9	13.5		32.9		9.2
**FE (mm)**	38.2		41.6			35.5	49.1		7.7	12.7	42.6
**Cusp to Cervix mesial lobe (mm)**	41.4		44.8	15.7	43.9	40.2	54.6		40.9		43.3
**Cusp to Cervix lateral lobe (mm)**	42.3		43.8	19.8	45.1	40.2		14.1	43.4	49.9	
**Total Samples**	14	6	12	9	11	14	17	7	22	13	22
**Cusp Sampled (all buccal)**	mesial	central	distal	distal	distal	mesial	mesial	distal	distal	mesial	mesial

Wear according to [51], the tooth biometrics DE, FD, FE are according to [52]. MHM = Malmö Museum.

Around 2000 bone specimens were identified to species from contexts dating from the early Neolithic to the early Middle Neolithic [17]. Over half of the determined specimens were from domestic cattle, with the remainder predominantly deriving from swine (*Sus* sp.), ovicaprids (*Capra* sp./*Ovis* sp.), and red deer (*Cervus elaphus)*. Of the cattle, sex determinations could not provide an interpretable dataset but the age profile of animals showed a culling of calves and young animals, which was interpreted as representing herd exploitation for meat. However, the presence of older animals was also reported, and interpreted as possibly indicating dairy production [17]. Unfortunately, no residue analyses have been performed on the hundreds of kilograms of Funnel Beaker ceramics recovered at the site.

While aurochs were present in Europe during the transition in Scandinavia, Neolithic cattle remains from certain areas including Scania and Zealand are domestic, as their wild counterparts in these areas went extinct many centuries prior [49–50]. Therefore, the mandibular teeth recovered from Almhov certainly represent domestic cattle. Unfortunately, due to conditions of preservation, we were only able to successfully sample teeth from six individuals. These animals were recovered from four EN I contexts, Features 35862, 19049, 25594, and Feature 6 from the initial test excavations (Table 1). No teeth were directly AMS dated, but all contexts from which the teeth derive date to the first phase of the Funnel Beaker Neolithic, the EN I, and represent the most secure contexts at the site (S1 Table) [17].

Care was taken to select teeth from a maximum number of individuals. Therefore, samples were selected from the same anatomical flank: the right side. The only exception was Tooth 35, which was a left M1 with similar wear and size to Tooth 4. While Tooth 35 and Tooth 4 derive from the same feature, the teeth had dissimilar patterns of mineralisation on complementary lobes, and were considered to derive from different individuals. This was confirmed by the data obtained in this study (see Results: Carbon and Oxygen), which indicates consistently dissimilar carbon isotope values along the length of the crown, ruling out a common individual of origin.

Teeth were first cleaned by abrading the surface using a diamond-tipped burr bit on a variable-speed rotary hand tool, removing all cementum and the outermost enamel surfaces. Samples were then drilled perpendicular to the axis of the tooth, starting at the cusp and proceeding to the cervix, leaving a ridge between samples and the distance of each sample from the ERJ was measured (S1 Fig). All teeth were at least in the process of mineralisation, and therefore the ERJ was discernable in all cases. Powdered enamel was then processed and analysed according to standard methods reported in-depth elsewhere [35]. Results were calibrated using laboratory and international standards, and output analytical error was determined to be ± 0.19‰ for ^δ18^O_SMOW_ (1*σ*) and ± 0.03‰ for ^δ13^C_VPDB_ (1*σ*).

**Fig 2:**
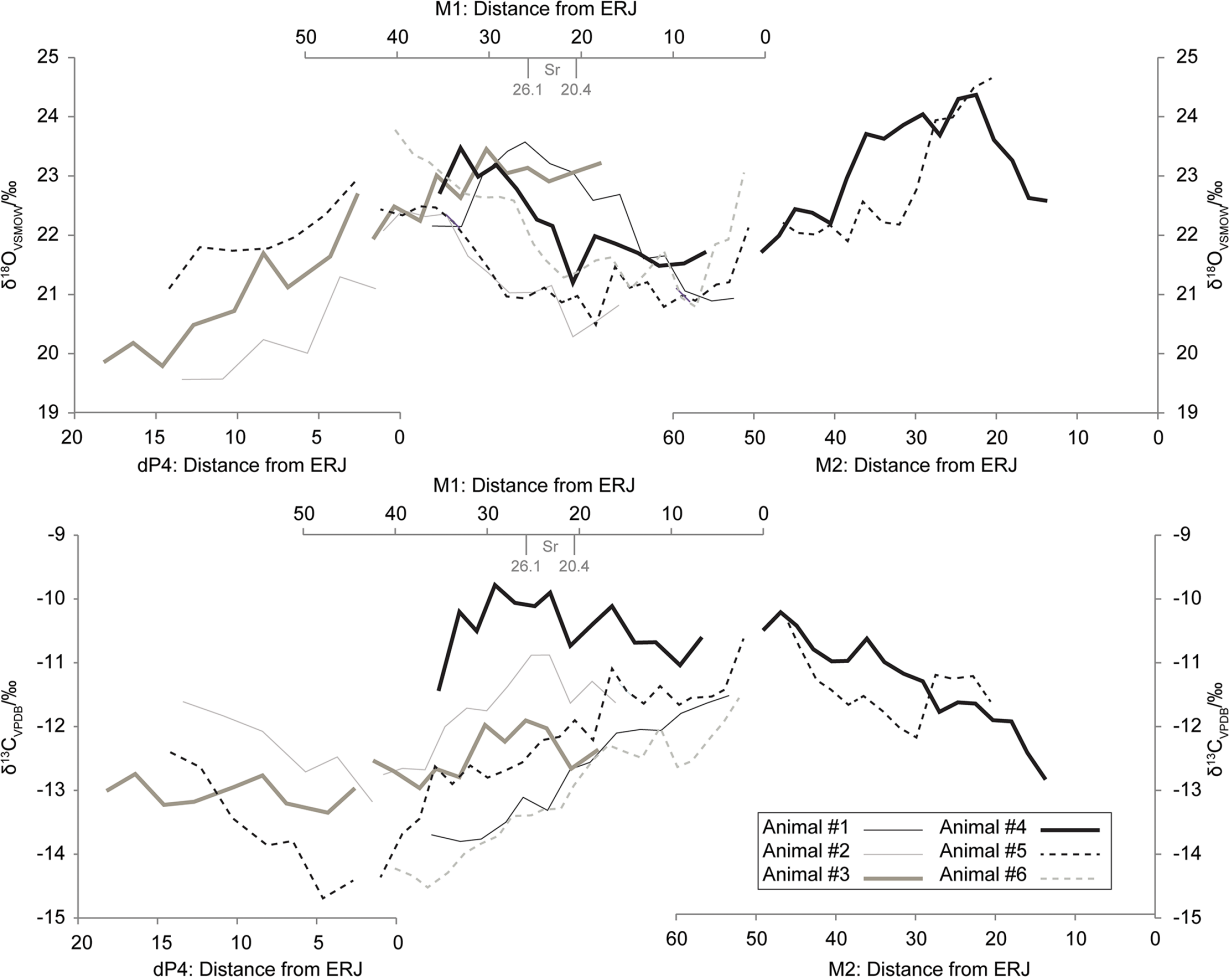
Oxygen and Carbon sequential sampling isotopic plots. Distances in mm, periods of developmental overlap eliminated for clarity.

Of the larger sample of teeth drilled for carbon and oxygen isotopic ratio analyses, the M1s from all six animals (Tooth numbers 2, 4, 6, 7, 10, and 35) were subsequently re-drilled for the strontium values in their tooth enamel. Teeth were neither bulk-sampled nor sequentially sampled down the length of the cusp, as the goal was not to ascertain potential transhumance or an average value. Instead, the teeth were sampled at a discrete point in the animal’s life in order to obtain a similar, snapshot view of the locality where the cow spent its first weeks and months. A further constraint on sampling was the fact that several of the teeth had incompletely mineralised portions closer to the ERJ. To accommodate this, a zone between 26.1 and 20.4 mm from the ERJ was sampled on each tooth and the particular zone of sampling is indicated in Fig 2. This range was chosen as it was the region on the six teeth which was most consistently close to the ERJ but in all cases also completely mineralised, as there was a degree of variation in the teeth in this regard. While there is some variation in the size of the teeth and their wear, this sampling strategy maximised the mass of the enamel sample, while at the same time mitigating as best as possible sources of variation in the source material. After drilling the enamel from the teeth, samples were prepared and analysed in the Laboratory for Archaeological Chemistry at the University of Wisconsin-Madison and the Department of Geological Sciences at the University of North Carolina-Chapel Hill using standard methodology reported in-depth elsewhere [48].

## Results

### Carbon and Oxygen

In all, 147 samples were analysed from three deciduous fourth premolars (dP4s), six M1s, and two M2s (Table 1, S2 Table). The eleven teeth derived from six animals. These data were used to build isotope curves from the raw data (Fig 2, S2 Table).

The spread of δ^13^C values between the animals is broad, encompassing a range of ~5‰ (Fig 2; -14.5‰ to -9.8‰ in the M1s). In conjunction with variation of peak values in δ^18^O (e.g. between Animal #1 and Animal #5), this may indicate year-to-year variation in climate, precipitation, and the diet of the animals. Further, δ^13^C values do not plateau in the M2 as in some populations of cattle but instead appear to vary seasonally. This makes identification of the point of inflection in the M1 which can indicate the onset of rumination [35] impossible to identify in this dataset. In all, this variation in δ^13^C values may indicate change in the composition of the fodder through the year. Only two in number, the M2s illustrate a probable offset in the timing of δ^18^O trends between Animals #4 and #5. While the data from the M2s do not contain complementary δ^18^O_max_ or δ^18^O_min_ values for direct comparison, the spring trend upwards in δ^18^O values from the two animals is offset by ca. 10mm from the ERJ.

We decided to include Tooth 35 from Animal #6, a left M1, to bolster the sample size and because of morphological dissimilarities with the other project samples. However, the Grant [51] wear stage of Tooth 35, while not the same as in Teeth 6 and 4 (Animals #2 and #3), is only one wear stage separated from each (Table 1). Nonetheless, the teeth are distinguishable both in their δ^13^C and δ^18^O profiles insofar as Tooth 35 had a δ^18^O curve intermediate between the two and much lower δ^13^C values than the other teeth. Given that molars from opposing sides of the mouth are morphologically mirror images of one another [53], Tooth 35 undoubtedly represents a different individual.

The most interesting result concerns the M1 data, which indicate birth in more than one season. Qualitative observation of the δ^18^O curves shows three groups of animals: Animals #2 and #5, Animals #4 and #6, and Animals #1 and #3. The curves from Animals #2 and #5 are the opposite to those from Animals #1 and #3, that is, they are approximately at their peak while their counterparts are at their minimums. Animals #4 and #6 are intermediate between the other two groups. There is some variation in the peak δ^18^O values, probably reflecting yearly climatic variation. Variation as a result of altitudinal change in residence can be ruled out on the grounds that Scania’s highest point is just over 200 metres above sea level, and at this latitude, any transhumance would effect negligible changes to the δ^18^O values [54].

Doubts concerning the constancy of the rate of mineralisation in M1s have been raised [35, 55–57]. This concern is in part based on Brown et al.’s [34] report that only the upper one-third of the M1 was mineralised at birth, implying that during the total developmental timeline of the tooth *in* and *ex utero* [34, 36], the remaining two-thirds developed in a period of only two to three months. If true, this would mean that there is a considerable acceleration in the pace of tooth mineralisation over the period of tooth development. In the M1 curves constructed here, even if the M1 from Animal #1 is estimated in unworn height at 40mm, three millimetres below the minimum values given by Legge [58], its δ^18^O_max_ and δ^18^O_min_ both fall in the lower two-thirds of the tooth. As this period must represent the six months between summer maxima and winter minima, there is no evidence for acceleration of mineralisation. Ultimately, these doubts stem from a lack of controlled experiments in modern cattle and the incongruence between tooth matrix deposition and maturation, which are important clarifications required of future research.

There are two main sources of error which could shift the distances of an individual sample value along the length of a tooth relative to a sample from a different tooth: variation in unworn overall length and variation in the period of development of an individual tooth. Caution must be taken as Balasse et al. [59] found that in sheep, the majority of variation in the placement of δ^18^O_max_ and δ^18^O_min_ values was due to variation in unworn tooth height. Unfortunately, there is no record of variation in unworn first molar height from early Neolithic cattle in southern Scandinavia although Legge [58] reported unworn crown heights ranging from 43 to 45mm in height in Bronze Age British M1s. Given that all the Almhov molars are at least slightly worn, and the maximum distance from the ERJ sampled in an M1 in this study was 42.6mm (Tooth 6, Animal #3), these values likely approximate the maximum unworn height of the M1s reported here. A difference of two millimetres in the unworn height of a M1 between 43 and 45mm in height effects at a maximum, 4.7% difference on the distance of an individual sample from the ERJ.

Similarly, Brown et al. [34] and Soana et al. [36] report that the M1s start forming at 140 days *in utero*, and are completed by the second or third month *ex utero*. This translates into 6.5 to 7.5 months of development in sum. A difference of one-month range in developmental timing therefore, at a maximum, has the potential to effect a 15.4% difference on the distance of an individual sample from the ERJ. Therefore, in all, the potential exists for a combined 20.0% margin of error on the values obtained as measured by distance from the ERJ.

**Fig 3:**
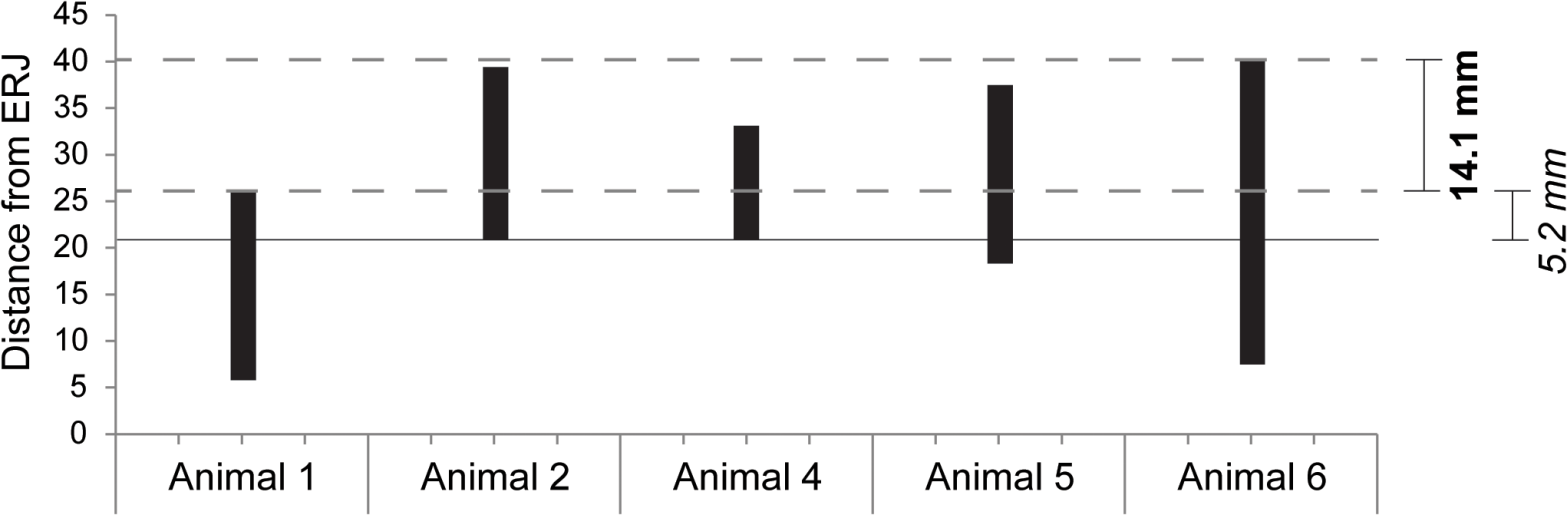
Maximum distance (mm) between summer maxima δ^18^O_max_ (bold) and least distance between an individual δ^18^O_max_ and δ^18^O_min_ (italic). Each bar represents δ^**18**^O_**max**_ at the top, and δ^**18**^O_**min**_ at the bottom. Animal #3 is excluded because its δ^**18**^O_**min**_ cannot represent the actual minimum of the seasonal curve.

Quantitatively, the absolute maximum spread of distance from the ERJ between summer maximum δ^18^O_max_ values and the absolute minimum spread between an individual δ^18^O_max_ and winter minimum δ^18^O_min_ (Fig 3) demonstrates that there is less separation between at least one δ^18^O_max_ and one δ^18^O_min_ (5.2mm) than between the largest spread of individual δ^18^O_max_ values (14.1mm). The null hypothesis that births are restricted to a single period of the year, as is expected for a cattle population in northern temperate environments [33], is rejected on these grounds. Cattle births at Almhov cannot be considered seasonal and took place in at least two, probably opposing seasons. If the maximum error of 20.0% is applied to minimize the distances between the δ^18^O maxima and maximize the smallest distance between δ^18^O maxima and minima the values become 11.28mm between maxima and 6.24mm between the closest maximum and minimum. Even with > 20% error, the null hypothesis that the cattle were all born in the same season can still be rejected.

It is necessary to note that while it appears that there is some variation in phase, that is, the millimetre distance between the δ^18^O_max_ and δ^18^O_min_ in individual teeth, and therefore probably the speed of mineralisation, the two teeth exhibiting the least distance between an individual δ^18^O_max_ and δ^18^O_min_, the M1s from Animal #1 and Animal #2, have phases that differ by only 1.8mm. This minor variation does not affect the result if applied in the simplest correction possible, by subtracting and adding this value respectively to the maximum error corrections (9.48 and 8.04mm).

Suckling and the timing of weaning have the potential to be contributory to δ^18^O values in herbivores given that water is obtained from the mother prior to birth and weaning [60–61]. However, such influences likely do not influence the data to any significant degree that would change our interpretations. As above, the two curves exhibiting the least distance between individual δ^18^O_max_ and δ^18^O_min_ values, Animals #1 and #2, have phases that only differ slightly, indicating little evidence of any change in ingested water source. Furthermore, all δ^18^O_max_ values and the aforementioned minimum distance between an individual δ^18^O_max_ and δ^18^O_min_ value fall from the middle to the upper half of the first molar. As mineralisation of the M1 proceeds mainly *in utero* [34, 36] from the crown, these data points are not influenced to any great degree by weaning as the animal was not yet born. Lastly, any contributory influence of a weaning signal would dampen summer seasonal increases in δ^18^O or decrease the overall curve values and this is not observed in these data.

Finally, the M1 of Animal #3 could only be sampled on approximately the highest two-thirds of the crown and drilling was aborted before the curve started trending down due to incomplete mineralisation. This animal serves to reinforce the results presented here as its curve qualitatively approximates that of Animal #1 and the two animals were likely born in the same season. Furthermore, its curve had not yet started trending down, indicating a later δ^18^O_max_ which only can serve to expand the maximum difference between maxima, and to further minimize the minimum difference between a single δ^18^O_max_ and a single δ^18^O_min_.

### Strontium

All animals except one had strontium isotope ratios within the usual range of variation for southern Scandinavia and the north European lowlands (Table 2) [47, 62], a region of geological and strontium isotopic similarity covering a considerable area stretching from the Netherlands to Poland. The values across the region are largely homogenous, so it is possible that these cattle came from elsewhere in the region, including possibly far afield, but we have no evidence in these data to suggest that they were anything but raised locally. Animal #5 has a slightly higher ^87^Sr/^86^Sr value relative to the other cattle which is outside the normal range for southern Scandinavia [47, 62]. This may indicate that this animal was moved to Almhov from another location, perhaps to the north, where higher ^87^Sr/^86^Sr values are recorded ca. 100km to the northeast [47–48]. However, Animal #5’s values may also reflect variation owing to a number of local and environmental factors, not least variations in underlying bedrock and drift cover, and in fact it is lower than a handful of published faunal samples from Denmark which are higher than the normal range [62]. Given this ambiguity, and as all other animals fall within the normal expected range, the data indicate that all animals were most likely raised locally.

**Table 2:** M1 Strontium Isotope Data.

Lab Number	Tooth Number	Animal Number	^87Sr^/^86Sr^	Distance from ERJ
F9561	2	1	0.710361	26.1–20.9
F9562	4	2	0.709054	25.2–20.9
F9563	6	3	0.710196	25.8–20.8
F9564	7	4	0.709925	24.8–20.9
F9565	10	5	0.711117	24.0–20.4
F9566	35	6	0.709660	23.9–20.5

## Discussion and Conclusions

We have presented evidence of cattle husbandry practice in the earliest Neolithic of southern Scandinavia. We have demonstrated that births did not occur in a single season in this population of cattle. Cattle births in more than one season are contrary to traditional cattle husbandry practices in northern Europe and also to the behavior of wild and feral bovids.

The data presented here mean two main things. First, breeding must have been artificially manipulated to produce calving and lactation throughout the year. Milk productivity in dairy cattle declines precipitously four or five months postpartum [27], so this manipulation of birth season is consistent with a strategy intended to maximize milk yield for year-round production. Secondly, multiple seasons of birth mean that increased fodder for the lactating cows must have been provided at suboptimum times of the year. This implies substantial and extensive planning and storage of fodder in order to ensure breeding at controlled times and to meet the dietary requirements of a lactating cow.

The faunal assemblage included juvenile and adult individuals but few very young calves. This mortality profile was interpreted as the result of exploitation of cattle for meat [17], as an idealised dairy production profile would indicate an immediate postpartum cull of very young calves [63]. However, the likelihood that Almhov was not a settlement, but rather a communal centre in only sporadic use, means that we should not expect to find the entire cattle herd—only those animals brought here for activities such as communal feasting accompanying mortuary rituals. A similar situation is found at other communal Neolithic sites, such as the somewhat later causewayed enclosure at Hambledon Hill in southern England where evidence for dairying is strong, but the majority of the cattle were around two years of age [64]. However, even if the entire herd was present at Almhov, the presence of juvenile and adult individuals does not discount dairying, as in some herds, calves are kept alive in order to encourage their mothers to let down milk [65–66].

Taken together, the faunal and isotopic data indicate an integrated, multiple-product system of cattle husbandry, geared towards providing both milk and meat throughout the year. While previous evidence for the consumption of milk from cattle has been identified on pot residues from the EN I in Sweden [15], the seasonality of birth in the cattle in this study confirms and underscores the primacy of dairying in the cattle husbandry regime in this earliest period of the Neolithic, not simply the incidental consumption of dairy products by Neolithic farmers. This emphasizes the importance and complexity of agriculture in southern Scandinavia from its very outset.

A regime this complex cannot represent the initial adoption of some agricultural traits by local hunter-gatherer populations, who would lack the skills, knowledge, experience, and even the vocabulary required to manage domestic livestock [14]. It comprises a fully formed technology of food production, one that must have taken humans a long time to develop. Almhov was used by some of the very first farmers in Sweden, so this development must have taken place somewhere else. Our findings therefore offer strong support for immigration as a major cause of agricultural origins in the region, with the immigrants bringing these sophisticated cattle management practices with them as part of their overall agricultural economy. Many archaeologists have argued for the gradual adoption of agriculture by native hunter-gatherers [14]. Recently, however, evidence has begun to emerge in support of migration [11, 14], and our results strongly support the immigration hypothesis.

## Supporting Information

S1 Fig Tooth 10, Animal #5 after sampling enamel for carbon and oxygen isotope ratios. (TIF)

S1 Table Animal, tooth, and feature numbers and dates from Almhov [17]. (TIF)

S2 Table Raw carbon and nitrogen isotope data. (XLSX)
