# Supplementary figures and images for: Cattle Management for Dairying in Scandinavia’s Earliest Neolithic
Source: PLoS One. 2015 Jul 6;10(7):e0131267. doi: 10.1371/journal.pone.0131267 (PMC4492493; doi:10.1371/journal.pone.0131267)

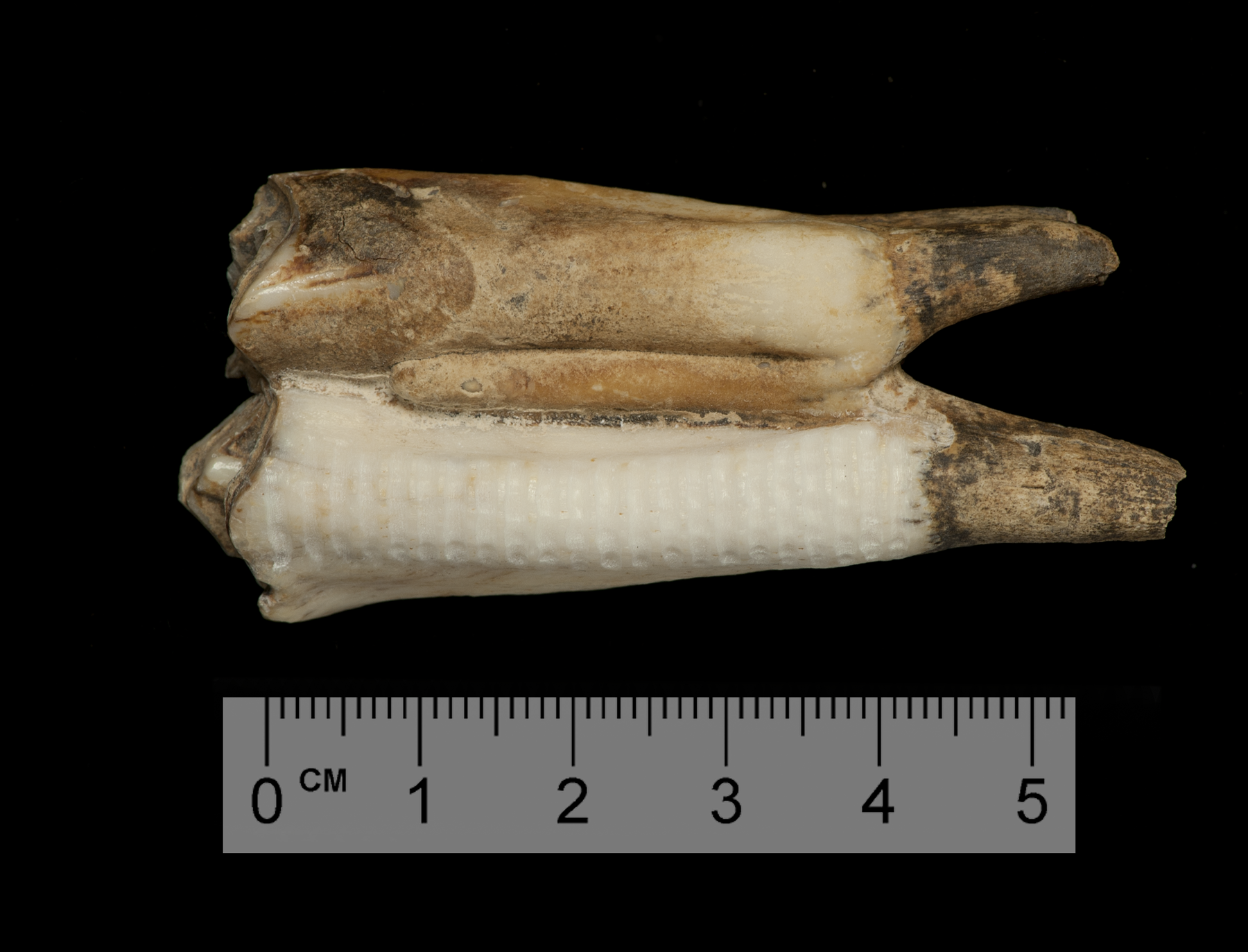

Supplement: S1 Fig — (TIF) [file pone.0131267.s001.tif]

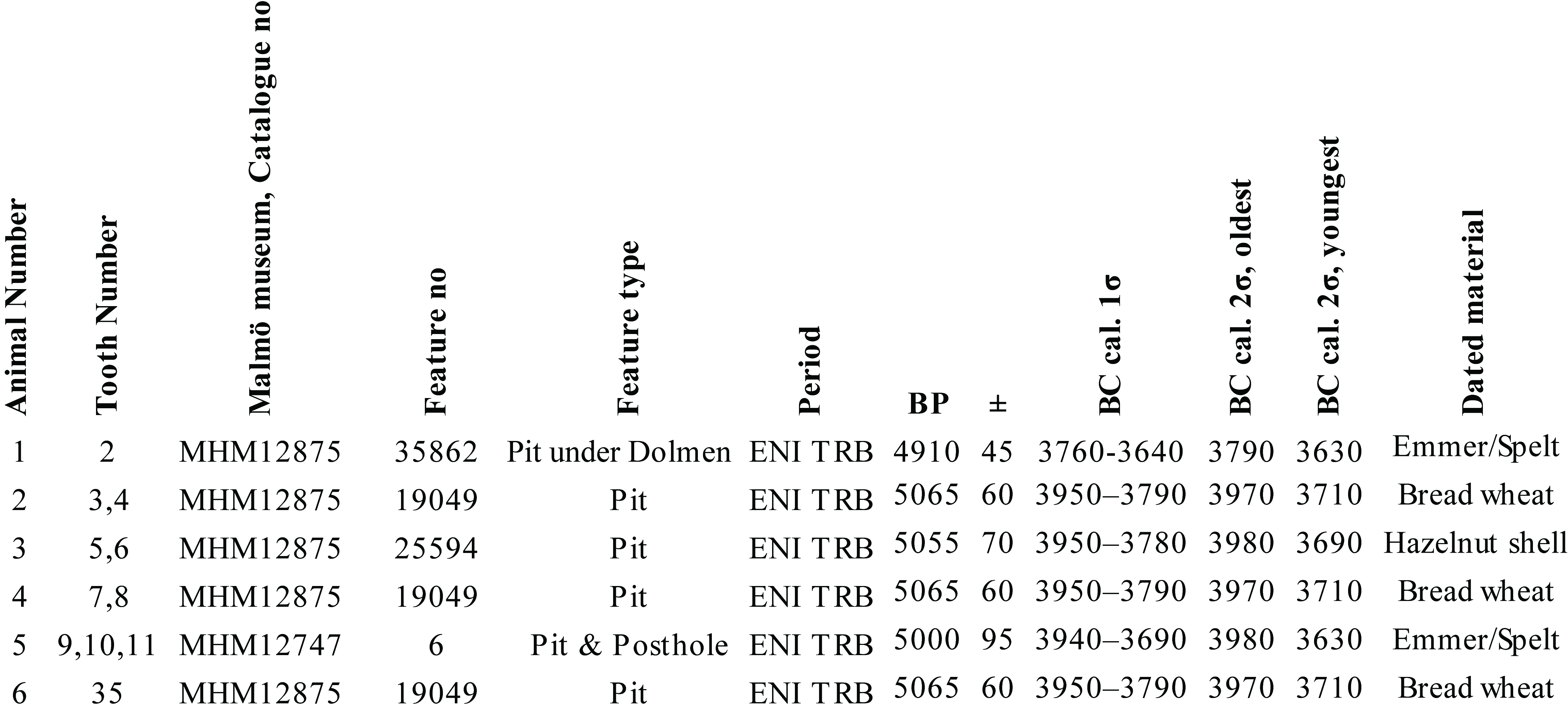

Supplement: S1 Table — (TIF) [file pone.0131267.s002.tif]
